# The Circular RNA Profiles of Colorectal Tumor Metastatic Cells

**DOI:** 10.3389/fgene.2018.00034

**Published:** 2018-02-09

**Authors:** Weiqin Jiang, Xingchen Zhang, Qinjie Chu, Sen Lu, Linfu Zhou, Xingang Lu, Chen Liu, Lingfeng Mao, Chuyu Ye, Michael P. Timko, Longjiang Fan, Haixing Ju

**Affiliations:** ^1^Cancer Biotherapy Center, The First Affiliated Hospital, School of Medicine, Zhejiang University, Hangzhou, China; ^2^Department of Agronomy, Institute of Bioinformatics, Zhejiang University, Hangzhou, China; ^3^Research Center for Air Pollution and Health, Hangzhou, China; ^4^Departments of Colorectal Surgery, The First Affiliated Hospital, Zhejiang University, Hangzhou, China; ^5^Medical Biotechnology Laboratory, Zhejiang University, Hangzhou, China; ^6^The 2nd Clinical Medical College, Zhejiang Chinese Medicine University, Hangzhou, China; ^7^Zhejiang Cancer Hospital, Hangzhou, China; ^8^Departments of Biology and Public Health Science, University of Virginia, Charlottesville, VA, United States

**Keywords:** colorectal cancer (CRC), metastasis, Circular RNA (circRNA), differential expressed circRNA (DEC), SW480 and SW620 cell line

## Abstract

Circular RNAs (circRNAs) have been reported that can be used as biomarkers for colorectal cancers (CRC) and other types of tumors. However, a limited number of studies have been performed investigating the potential role of circRNAs in tumor metastasis. Here, we examined the circRNAs in two CRC cell lines (a primary tumor cell SW480 and its metastasis cell SW620), and found a large set of circRNA (2,919 ncDECs) with significantly differential expression patterns relative to normal cells (NCM460). In addition, we uncovered a set of 623 pmDECs that differ between the primary CRC cells and its metastasis cells. Both differentially expressed circRNA (DEC) sets contain many previously unknown putative CRC-related circRNAs, thereby providing many new circRNAs as candidate biomarkers for CRC development and metastasis. These studies are the first large-scale identification of metastasis-related circRNAs for CRC and provide valuable candidate biomarkers for diagnostic and a starting point for additional investigations of CRC metastasis.

## Introduction

Circular RNAs (circRNAs) are covalently closed, single-stranded transcripts produced from pre-mRNA back-splicing. They were first discovered more than 20 years ago and were initially thought to be the by-products of aberrant splicing with little functional potential (Nigro et al., 1991; Cocquerelle et al., 1992, 1993; Capel et al., 1993; Pasman et al., 1996; Zaphiropoulos, 1997). Assisted by the increased availability of high-throughput sequencing technology and bioinformatics softwares to identify potential circRNAs, the expression of circRNAs were shown to be widespread in metazoans such as humans (Salzman et al., 2012; Jeck et al., 2013; Memczak et al., 2013), mouse (Memczak et al., 2013), nematode (Memczak et al., 2013), fruit flies (Westholm et al., 2014), and plants such as rice (Lu et al., 2015; Ye et al., 2015), Arabidopsis (Wang et al., 2014; Ye et al., 2015), barley (Darbani et al., 2016), wheat (Wang et al., 2017). Most circRNAs originate from exons of protein-coding genes and can consist of one or multiple exons, while some circRNAs also arise from introns, intergenic regions, non-coding RNA (ncRNA) loci, and other portions of the genome (Jeck et al., 2013; Memczak et al., 2013). Multiple circRNAs can generate from a single gene locus through alternative back-splicing events (alternative circularization) (Zhang et al., 2016; Ye et al., 2017). Generally, circRNA biogenesis involves the canonical spliceosomal machinery (Starke et al., 2015; Chen, 2016) and is dependent upon various cis-regulatory elements such as repetitive complementary sequences (Liang and Wilusz, 2014; Zhang et al., 2014) and trans-acting factors such as Mbl (Ashwal-Fluss et al., 2014), QKI (Conn et al., 2015). Additionally, conserved expression of circRNAs generated from orthologs was also observed in closely related species (Rybak-Wolf et al., 2015; Ye et al., 2015; Ebbesen et al., 2016).

While the expression of a majority of circRNAs is often lower than that of their linear isoforms, some circRNAs, whose existence has been experimentally confirmed, are expressed higher than their linear isoforms (Ashwal-Fluss et al., 2014; Rybak-Wolf et al., 2015). And some circRNAs have been reported to function as miRNA sponges (Hansen et al., 2013a; Memczak et al., 2013; Piwecka et al., 2017) and regulate transcription of host genes by promoting transcription (Zhang et al., 2013; Li Z. et al., 2015), splicing competition (Ashwal-Fluss et al., 2014), RBP interaction (Ashwal-Fluss et al., 2014; Conn et al., 2015). Recent studies have also demonstrated that some circRNAs may also have important functions in regulating cellular development (Szabo et al., 2015) or in the initiation or progression of some diseases (Guarnerio et al., 2016). To date, many circRNAs worked as potential ceRNAs or biomarkers related to human cancers have been identified (Kulcheski et al., 2016). For example, miR-7 has been reported to target several genes involved in cancer development as well as to be suppressed by ciRS-7 (Hansen et al., 2013b), indicating the potential relationship between circRNAs and cancer.

Metastasis is the major cause of death for cancer patients (Chaffer and Weinberg, 2011). The tumor invasion-metastasis cascade is a stepwise and multi-stage process which requires tumor cells to survive in the circulation, extravasate, and colonize distant sites (Jin K. et al., 2017). The general processes of metastasis are similar among most tumor types, although metastasis to different tissues appears to require distinct sets of regulators and/or host micro-environments. Many key genes involving in tumor metastasis have been identified (Jin K. et al., 2017) including protein-coding genes and non-coding genes such as miRNAs (Ramaswamy et al., 2003; Alečković and Kang, 2015; Jin H. et al., 2017; Weyden et al., 2017).

The colorectal cancer (CRC) pathway in KEGG (Kyoto Encyclopedia of Genes and Genomes, www.kegg.jp) encompasses a diverse group of pathways (such as the TGF-beta, Wnt/beta-catenin and Notch pathways) during the progression from a colorectal epithelial cell to primary tumor cells and their subsequent proliferation in metastatic colonization. In addition to protein-coding genes, many non-coding genes have been implicated in CRC progression, including miRNAs and long non-coding RNAs (Bachmayr-Heyda et al., 2015; Wang J. et al., 2015). For example, 13 oncogenic miRNAs are up-regulated and 20 miRNAs are down-regulated in CRC tumor tissues (Wang J. et al., 2015). There also are dozens of CRC-related circRNAs that have been identified (Bachmayr-Heyda et al., 2015; Li Y. et al., 2015; Xie et al., 2016; Yang et al., 2016; Zheng et al., 2016). By comparing colorectal tumor cells and normal tissues, CRC-specific circRNAs have been identified in CRC tumors (Bachmayr-Heyda et al., 2015; Li Y. et al., 2015; Zheng et al., 2016; Hsiao et al., 2017; Zhang et al., 2017; Zhu et al., 2017) and it has been possible to correlate circRNA abundance with CRC proliferation. Relatively few non-coding circRNAs have been identified that specifically relate to metastasis of CRC and other cancers (Li Y. et al., 2015; Hsiao et al., 2017; Jin K. et al., 2017). Therefore, there is a clear need for broader investigation of the potential role of circRNAs in tumor metastasis.

Over 100 CRC cell lines have been developed and used in diverse studies of CRC (Spitzner et al., 2010; Adler et al., 2014; Medico et al., 2015). The SW480 and SW620 cell lines were established from a male patient with CRC (Leibovitz et al., 1976). The SW480 cells are derived from the primary tumor, whereas the SW620 cells are derived from a lymph node metastasis taken from the same individual at the time of a second laparotomy; the patient received no intervening chemotherapy, when recurrent cancer with liver and mesenteric lymph node metastases was discovered (Provenzani et al., 2006). The SW480 colon carcinoma cells, and their relative lymph node metastatic SW620 cells, have different metastatic abilities (Bergmann-Leitner and Abrams, 2000; Hewitt et al., 2000; Provenzani et al., 2006), with the growth advantage and tendency to autonomous clone growth of SW620 cells being three-fold higher than SW480 cells (Provenzani et al., 2006). Consequently, the SW620 cells have been routinely used to find metastasis-associated genes of colorectal carcinoma (Provenzani et al., 2006) or CRC-associated genes (Adler et al., 2014; Bachmayr-Heyda et al., 2015; Paschall et al., 2016; Rasmussen et al., 2016; Xie et al., 2016).

In this study, we compared the circRNAs and mRNAs from the metastatic cells (SW620) and their primary colon carcinoma cells (SW480), with normal colorectal cells (NCM460) as control. Our analysis revealed a unique circRNA expression profile in the SW620 cell lines relative to SW480 and NCM460 cell lines, suggesting that circRNAs could be used as molecular indicators of cancer progression.

## Results

### High metastatic rate of the CRC metastatic SW620 cells

**Figure 1:**
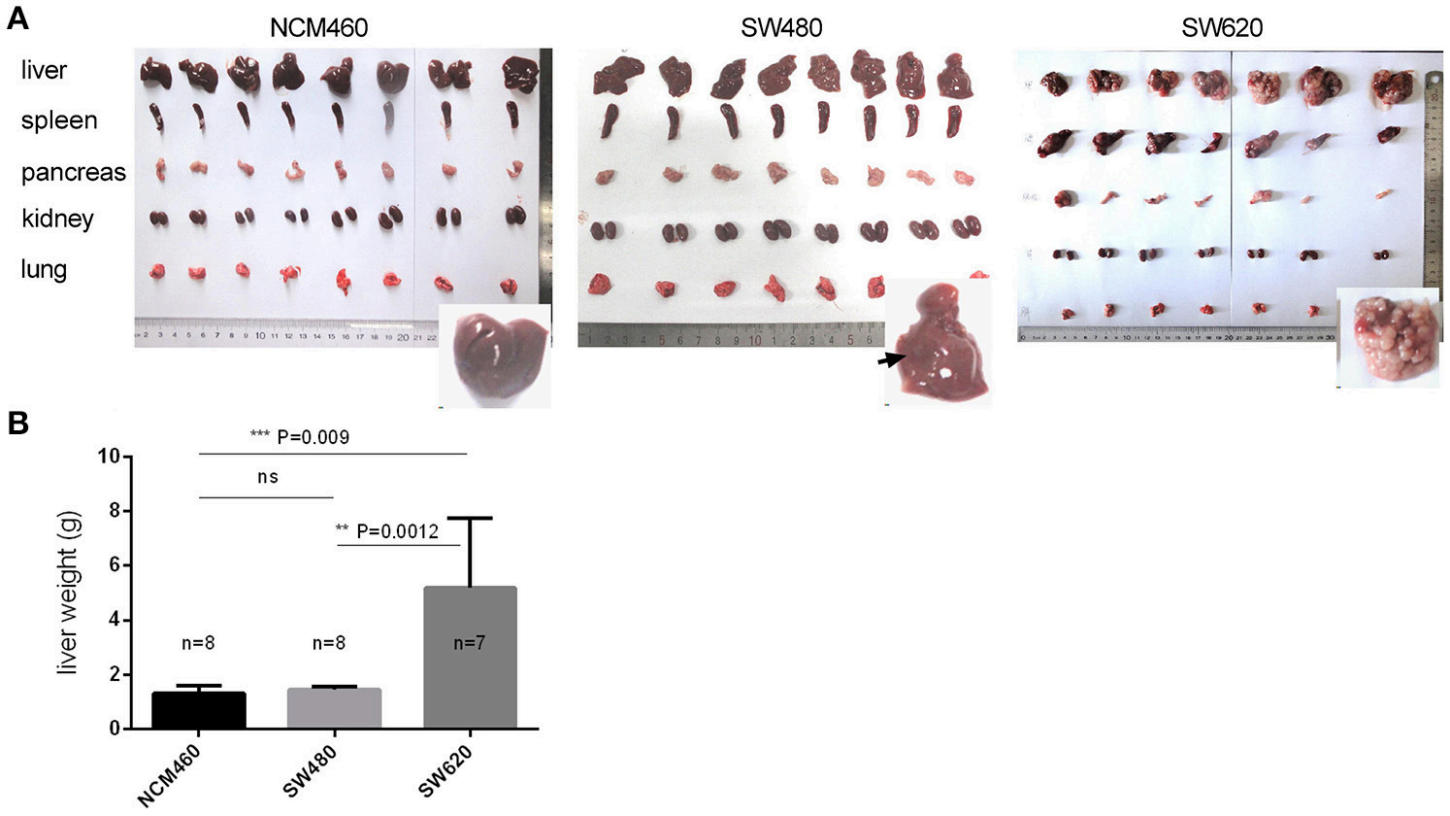
Tumorigenic and metastatic potential of CRC metastatic cell line (SW620) and its primary tumor (SW480) and normal (NCM460) cell lines. **(A)** Phenotypes of five tissues of nude mice by intrasplenic (IS) injections. **(B)** Statistical significance for the mean weights of livers in the three cell groups.

In order to confirm the clonal status and differential phenotype of the SW620 and SW480 cell lines used in this study, we performed a preliminary experiment using nude mice by intrasplenic (IS) injections to induce tumor development (see section Materials and Methods). Tumors were visible in the liver, spleen, and pancreas (Figure 1A, Table S1) following injection of the CRC cells. Numerous liver metastatic nodules were observed in mice injected with SW620 cells after 4 weeks. In contrast, no or very few nodules were detected in mice injected with NCM460 or SW480 cells (Figure 1A). In the SW620 group, tumors were visible in 7/7 livers, and all of the tumors weighed more than 2.0 g. The mean weight of liver tumors was significantly higher than that of other tumor groups at the same developmental stage (Figure 1B). Taken together, our findings indicate that the tumorigenic and metastatic potential of SW620 cells is higher than that of other cell lines, consistent with previous descriptions of these cells (Bergmann-Leitner and Abrams, 2000; Hewitt et al., 2000; Provenzani et al., 2006).

### Sequencing and quality control of circRNA-Seq and mRNA-Seq data

Circular RNA and mRNA were extracted from the metastatic SW620 cells, their primary SW480 colon carcinoma cells, and normal colorectal cells (NCM460), and sequenced (details in section Materials and Methods). Three biological replicates were performed. For each biological replicate, about 25 and 9 Gb circRNA and mRNA data were generated, respectively (Table S2).

FastQC and fastx-toolkit (see section Materials and Methods) were used to check and control the quality of the sequencing reads, respectively. Principle component analysis (PCA, details in section Materials and Methods) of gene expression showed that the three individual replicates of circRNA-Seq and RNA-seq data for each cell line clustered together, indicating that the samples were highly reproducible (Figure S1). In general, based upon our analysis, the data generated by this study are of high quality and therefore we undertook for following analysis.

### A significantly larger number of circRNAs was identified in normal cell line than that from the two CRC lines

Using the previously described CIRI2 circRNA prediction tool (Gao et al., 2017), we were able to identify 33,962 unique circRNAs from our three cell lines (Tables S3, S4). Of the 33,962 unique circRNAs, 25,042 (73.7%) were identified previously in other studies (Chen et al., 2008, 2016; Ghosal et al., 2013; Glazar et al., 2014; Table S3) and 8,920 were newly identified by this study. Of the 33,962 unique circRNAs, 28,340 (83.4%) appear to be derived from protein-coding exons, which is similar to the results of previous studies (Zheng et al., 2016).

**Figure 2:**
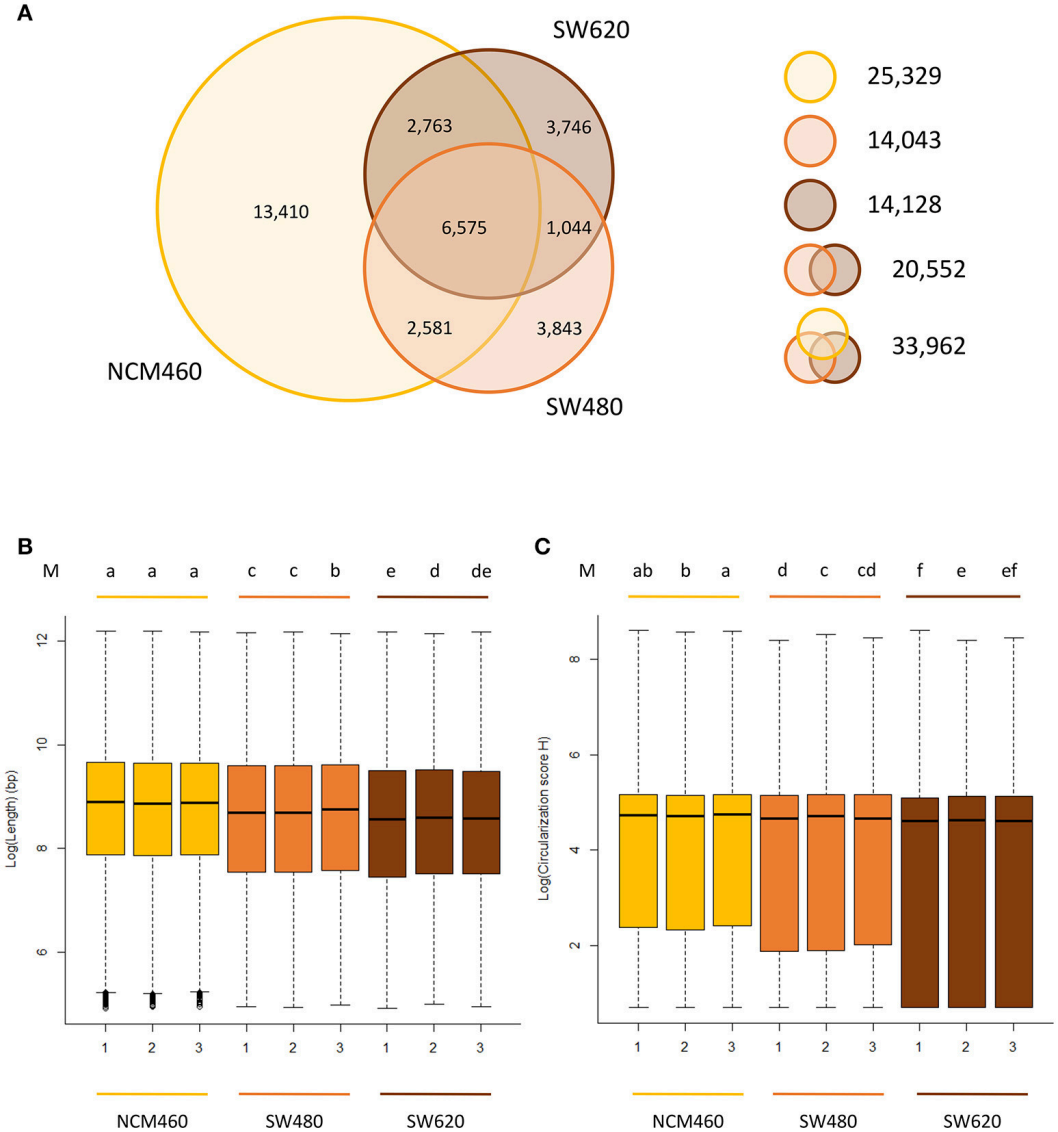
Characteristics of circRNAs from the CRC lines relative to normal line. **(A)** Number of circRNAs identified in three cell lines. Yellow, orange, brown circles represent the quantity of circRNAs in NCM460 (normal), SW480 (primary CRC), SW620 (metastasis CRC). **(B)** Genomic lengths of all circRNAs identified in three cell lines. **(C)** The circularization score *H* of reverse complementary matches in flanking introns of circRNAs identified in three cell lines. “M” in top of **(B,C)** are the results of LSD test with Bonferroni correction (alpha = 0.01) using the average value of lengths and score *H* data, respectively. Lowercase letters such as a, b, c, d represent the arrangement of mean values from high to low, and different letters represent the significant difference at the 99% level of confidence.

Approximately the same number (~14,000) of circRNAs were identified from both of the two CRC lines, while a significantly larger number (25,329) of circRNAs were identified from the normal cell line (Figure 2A, Table S3) despite the fact that it had the lowest raw data amount (66.7 Gb) among three cell lines (Table S2). Of the circRNAs identified from two CRC cell lines, only 20,552 unique circRNAs were found.

Of the 25,329 circRNAs from normal cell lines (NCM460), 13,410 circRNAs were only found in the normal cell lines but not the two CRC cell lines. In contrast, much fewer, about 3,800 specific circRNAs, were identified in each CRC cell line suggesting that circRNAs may be expressed significantly lower in CRC cells relative to their normal cells.

### Unique characteristics of circRNAs in CRC lines relative to the normal line

The circRNAs from two CRC cell lines have some unique characteristics when compared to those identified in the normal cell lines. For example, the sizes (genomic lengths) of the circRNAs are significantly smaller than those from the normal line (Figure 2B, *P*-value < 2.2e-16 by Wilcoxon rank-sum test). We also found that in the two CRC lines, the genomic lengths of circRNAs from metastasis line are much shorter than those from their primary cell line (Figure 2B). Circularization score *H* (Ivanov et al., 2015) was used to measure the reverse complementary matches of the flanking introns of circRNAs identified from three cell lines. A significantly lower value of score *H* in the flanking introns of circRNAs from CRC lines than in normal lines was observed (Figure 2C, *P*-value < 2.2e-16 by Wilcoxon rank-sum test). It has been reported that reverse complementary sequences of flanking introns, contribute to circRNA biogenesis (Ivanov et al., 2015). Usually, the circularization score *H* of reverse complementary sequences are much higher than normal sequences. Therefore, compared to the normal line, circRNAs may be more likely to be generated by other biogenesis mechanisms rather than reverse complementary sequences in CRC lines, in which predicted circRNAs have significant lower score *H* of flanking introns.

### 2,919 differential expressed circRNAs (DECs) between the CRC cells and their normal cells

**Figure 3:**
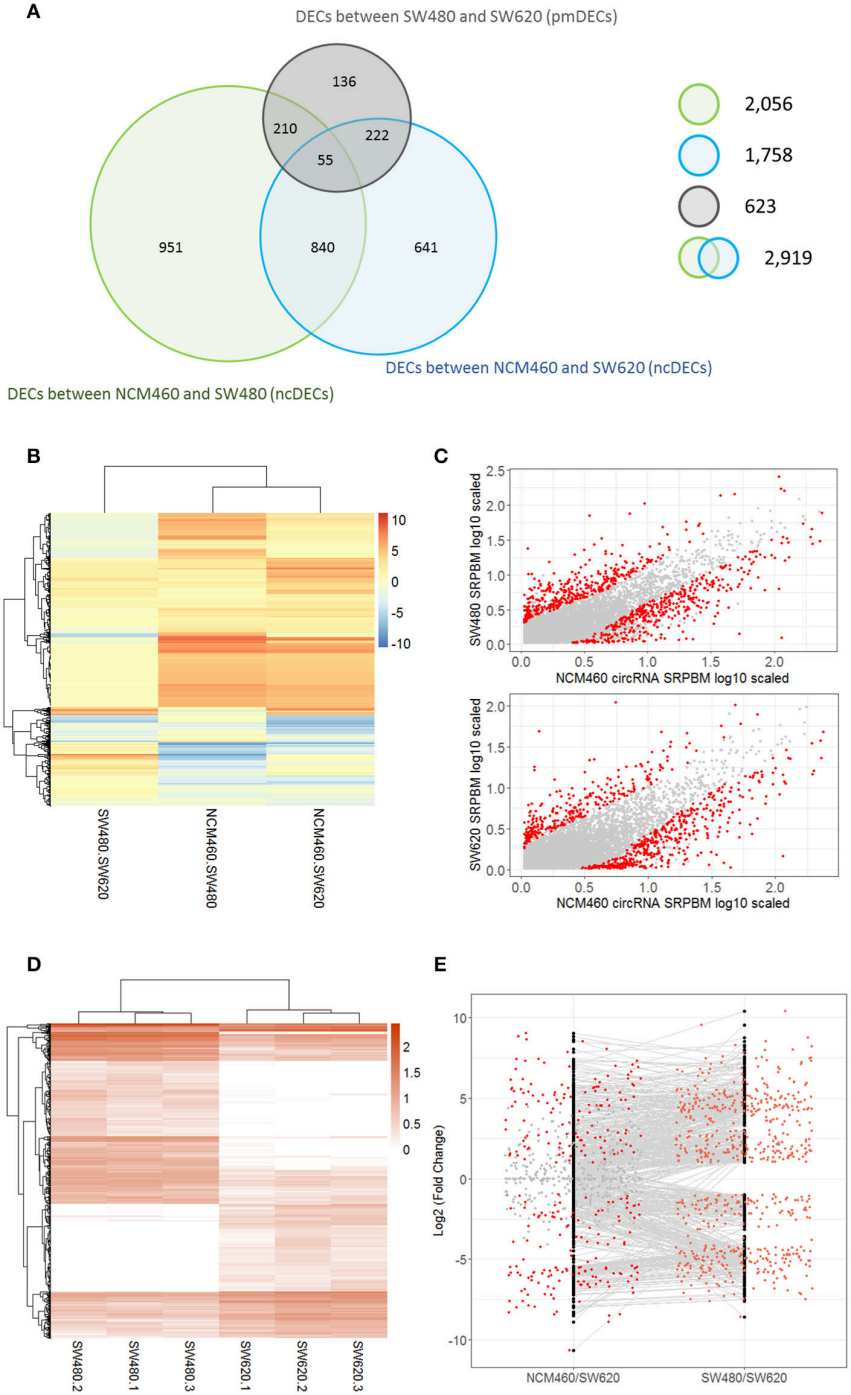
Number and expression patterns of differential expressed circRNAs (DECs) between the CRC lines (SW460 and SW480) and NCM460. **(A)** Number of DECs in different groups. Green, blue, gray circles represent the quantity of DECs between NCM460 and SW480, NCM460 and SW620, SW480 and SW620, respectively. **(B)** Clustered heatmap of 2,919 ncDECs, with columns representing different circRNAs, and rows representing fold-changes between the corresponding two cell lines. **(C)** Normalized expression values (SRPBM) of circRNAs in NCM460 vs. SW480, NCM460 vs. SW620, respectively. Red and gray points represent significantly differential expressed and non-significantly differential expressed circRNAs, respectively. **(D)** Clustered heatmap of 623 pmDECs, with columns representing different circRNAs, and rows representing three biological replicates of SW480 and SW620. **(E)** Expression fold-changes on log-scale of 623 pmDECs in SW480 vs. SW620 (right) and NCM460 vs. SW620 (left). Orange points represent DECs in SW480 vs. SW620. Red and gray points represent DECs and non-DECs in NCM460 vs. SW620.

A total of 2,919 unique DECs were identified between the CRC cells (SW480 or SW620) and their normal cells NCM460 (termed as ncDECs) in this study (Figure 3A, Table S4). Among these unique DECs, there are 2,056 DECs between the SW480 and NCM460, and 1,758 DECs between the SW620 and NCM460 (Figure 3A).

Some interesting results could be found from the expression patterns of 2,919 ncDECs (Figure 3B, Table 1). For example, 840 have similar expression levels in two CRC lines but are significantly decreased (709, Table 1, pattern NO. 1) or increased (131, Table 1, pattern NO. 7) compared with normal cell lines. Fifty-five (Table 1, pattern NO. 11–16) of 2,919 ncDECs not only differentially expressed between cancer and normal cell lines, but also differentially expressed between primary and metastasis cancer cell lines. The 895 mentioned above are common between the two ncDEC sets (i.e., the overlap of blue and green circles in Figure 3A, the expression of which were shown in Figure 3C). In addition, pattern NO. 2 and NO. 4 showed 32.6% circRNAs differentially expressed between primary cancer and normal cell lines; pattern NO. 3 and NO. 5 showed 22.0% differentially expressed between metastasis cancer and normal cell lines.

**Table 1:** Detailed express patterns of the 2,919 DECs in the two CRC cell lines (SW480 and SW620) relative to normal line (NCM460).

**Pattern NO**.	**Expression pattern^a^**	**Number**	**Percentage (%)**
1	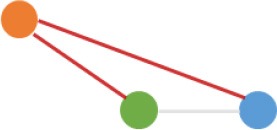	709	24.3
			
2	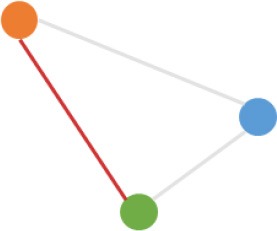	675	23.1
3	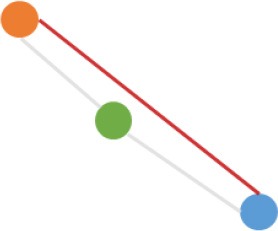	419	14.4
4	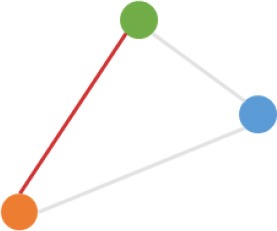	276	9.5
5	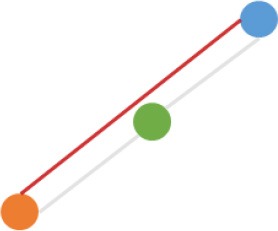	222	7.6
6	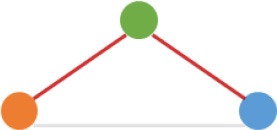	131	4.5
7	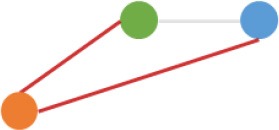	131	4.5
8	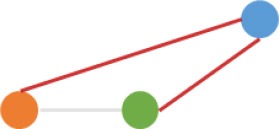	123	4.2
9	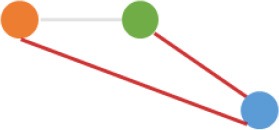	99	3.4
10	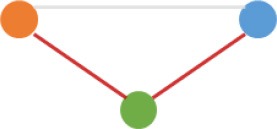	79	2.7
11	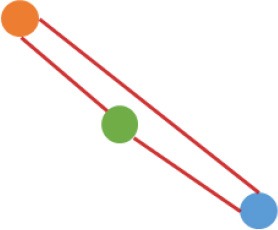	20	<1.0
12	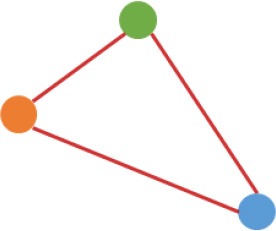	12	<1.0
13	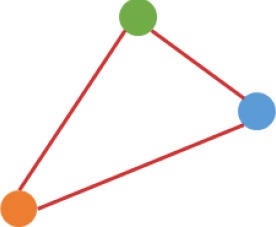	9	<1.0
14	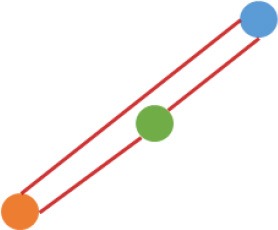	8	<1.0
15	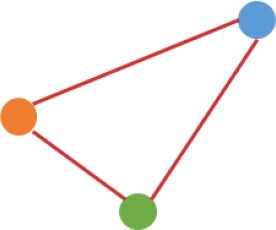	4	<1.0
16	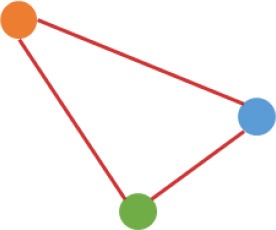	2	<1.0

a *Orange, green, blue circles represent expression of circRNAs in NCM460, SW480, SW620 cell lines, respectively. Color-filled circles at different height represent different expression levels of circRNAs in the corresponding cell lines. Red links between two circles represent significantly differential expression between circRNAs in the corresponding cell lines, while gray links means no change in expression. For example, pattern NO. 2 means, circRNAs from NCM460 significantly increased expressed compared with that from SW480, but there is no significantly differential expression between circRNAs from NCM460 and SW620, as well as circRNAs from SW480 and SW620*.

### 623 DECs between the metastatic SW620 and its primary SW480 cells

A total of 623 DECs were identified (FDR < 0.05 and fold-change >= 2) between the metastatic SW620 and its primary SW480 cells (termed as pmDECs; Figure 3A, Table S4). Among these 623 pmDECs, there are 487 (78.2%) that are common with the 2,919 ncDECs, of which 277 are also differentially expressed between NCM460 and SW620, and 265 are differentially expressed between NCM460 and SW480. Only 55 ncDECs (Table 1, pattern NO. 11–16) are differentially expressed in all three cell lines, which indicates that those circRNAs may involve in the progression of cancer.

Of the 623 pmDECs, 348 are down-regulated and 275 up-regulated in the SW620 line (Figures 3D,E). Many circRNAs showed no detectable level of expression in SW620 cells despite being highly expressed in SW480, or no expression in SW480 but highly expressed in SW620 (Figure 3E). For example, circRNAs from two genes, GLI3 (10 circRNAs) and RAPGEF5 (8 circRNAs), are down-regulated in SW620 (or with no detectable level of expression) relative to SW480. Meanwhile, some circRNAs are expressed in SW620 such as circRNAs from SLC22A3 locus (6 circRNAs) but not in SW480.

Twenty-eight of the 623 pmDECs were generated from 24 genes that are included in the cancer Gene Census (http://cancer.sanger.ac.uk/census; Table S5). Both significantly up- and down-regulated expression of these 28 circRNAs in SW620 cell lines relative to SW480 cell lines were observed. Similarly, the expression patterns of the genes from which they derive also have the same expression patterns in the SW620 relative to the SW480 cell lines, with the exception of two genes (GPHN and SPECC1). Each of the 24 genes had multiple loci generating circRNAs, including four genes (NFIB, ETNK1, SPECC1, ARHGAP26) which gave rise to two pmDECs, respectively (Table S5).

### High positive relationship of expression between 277 pmDECs and their host genes

**Figure 4:**
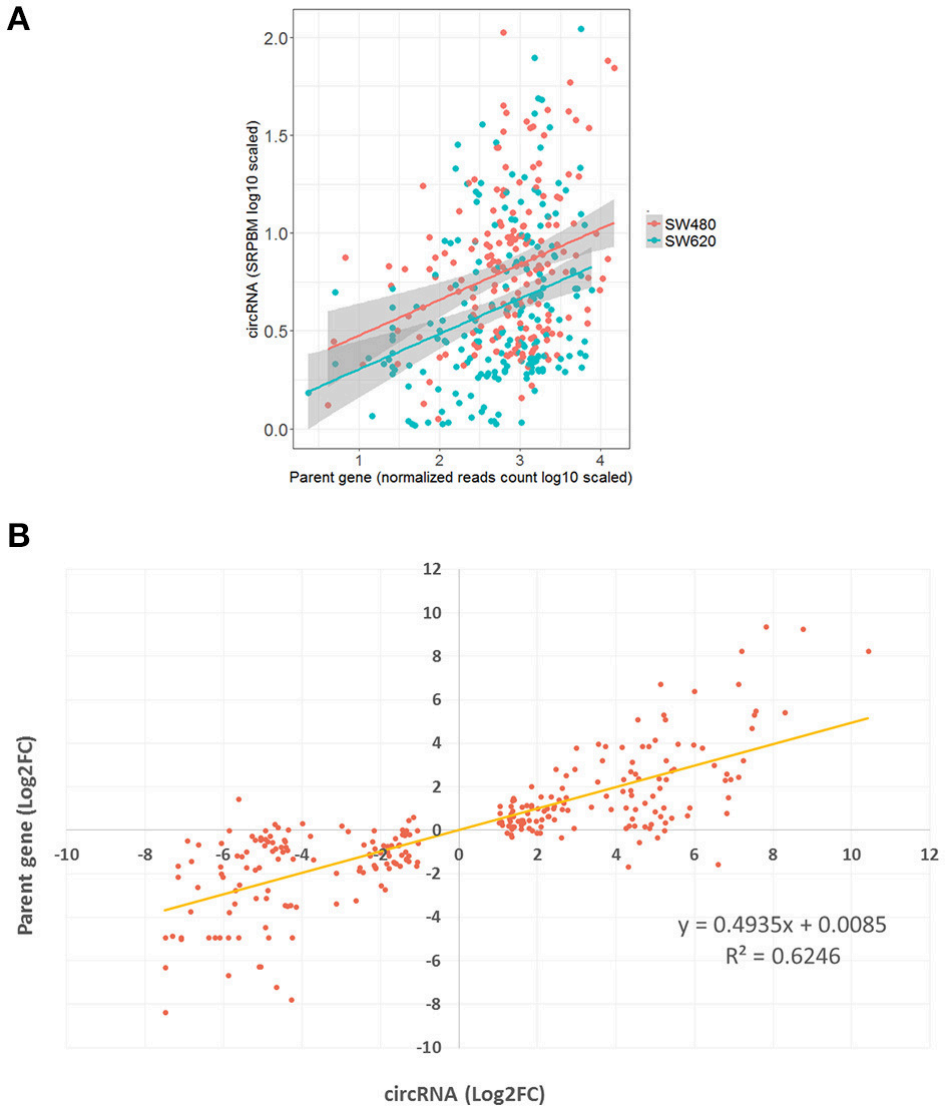
Positive relationship of expression between pmDECs and their host genes. **(A)** Expressions on log-scale of the 277 pmDECs and their host genes from SW480 and SW620 cell lines. **(B)** Equation of linear regression of fold-changes on log2-scale of the 277 pmDECs and their host genes between SW480 and SW620.

CircRNAs that are significantly differentially expressed between primary tumor cell and metastatic tumor cell, and also differentially expressed between normal cell and metastatic tumor cell, might be related to metastasis processes. A total of 277 DECs fell into this category and were used to investigate the relationship between levels of circRNA expression and expression of their host genes. We found a positive correlation between the expression of the 277 pmDECs and their host genes (Figure 4A; i.e., more highly expressed circRNAs have more highly expressed host genes in SW620 relative to SW480). The 277 pmDECs between the SW620 and SW480 were further used to plot expression fold-changes with their host genes (Figure 4B). The results indicate that the DECs between the metastatic SW620 and its primary SW480 cells have a highly positive expression correlation with their host genes (*R*^2^ = 0.62, *P* < 2.2e-16, Figure 4B). Based on the expression fold-changes of the ncDECs identified from the two CRC cell lines and their host genes, a positive correlation is also observed (Figure S2).

### Metastasis-related genes generating unique circRNAs

Many circRNAs were identified from metastasis-related genes (Ramaswamy et al., 2003; Jin H. et al., 2017; Weyden et al., 2017) or from genes that are involve in regulating miRNAs that target metastasis-related genes (Table S6). For example, it is reported by Li J. et al. (2015) that miR-96 can significantly lower the expression of Foxo3 which is associated with lymph node metastasis. We found at least 49 pmDECs that could serve as sponges for miR-96 or other miRNAs that target Foxo3 (Presentation 1, Table S7). The down-regulation of such circRNAs in SW620 would be expected to result in an up-regulation of miR-96 and down-regulation of Foxo3 (1.64-folds, *P* = 8.0e-27). As in the case of Foxo3 regulation, many complex circRNA-miRNA-mRNA interactions were predicted (Presentation 1, Table S7).

Another example is the SMURF2 network. SMURF2 has a key role in Ubiquitin E3 ligase regulation of the TGF-beta pathway (Jin K. et al., 2017) and was found significantly down-regulated (four-fold, *P* = 1.5e-246 by Wald test in DESeq2 package) in SW620 metastatic cells relative to SW480 primary tumor cells (Table S4). Two pmDECs from the SMURF2 were detected in our studies, both of which were also significantly down-regulated in SW620 (13.5-fold, *P* = 8.3e-7 and 11.9-fold, *P* = 4.4e-7 by Wald test in DESeq2 package) based on their junction spanning reads. These two pmDECs are up-regulated in SW480 compared with NCM460 but not differentially expressed in NCM460 and SW620.

## Discussion

### Candidate circRNAs involved in CRC development and metastasis

We have analyzed the circRNAs in two CRC cell lines, primary tumor cell lines and their metastasis derivative cell lines. We showed that a large number of circRNAs are present in tumor and metastatic cells that have significantly different expression levels relative to normal cells (2,919 ncDECs), including 895 ncDECs that are significantly differentially expressed in both NCM460/SW480 and NCM460/SW620 pairings. We also found a group of 623 pmDECs that are differentially expressed in primary cells compared to its metastasis cell. Among these are 277 pmDECs that are also differentially expressed between NCM460 and SW620. The expression patterns of these 277 pmDECs are unique among the three cell lines and could potentially be involved in the metastasis processes. In the sets, only about 180 circRNAs are in common with CRC-related circRNAs identified in previous studies (Table 2) and therefore, these circRNAs constitute a new large pool of candidate circRNAs that could serve as biomarkers for CRC development and metastasis. To our knowledge this is the first large-scale identification of metastasis-related circRNAs for CRC.

**Table 2:** Summary of putative CRC-related circRNAs identified by this study.

**Number of circRNAs^a^**	**Identified by**
2,870	Only this study
27	CRC biomarkers by this study
1	This study and Wang X. et al., 2015
3	This study and Bachmayr-Heyda et al., 2015
42	This study and Li Y. et al., 2015
104	This study and Zheng et al., 2016

a *See Tables S4, S9 for detailed circRNA information, respectively*.

Interestingly, circRNA circ-001569 previously reported to be involved in CRC (Xie et al., 2016) was not found among our circRNAs. However, we did identify at least 40 different circRNAs (3 DECs) that arise from the same ABCC1 locus that gives rise to circ-001569. Alternative splicing is a major contributing factor to the formation of circRNAs, and we believe that the different isoforms of circ-001569 identified in our experiments are of importance to CRC development and metastasis.

### Characteristics of circRNAs from CRC cells

In this study, we compared the circRNAs from normal and CRC cells and found several features which appear to be in common for CRC circRNAs. First, our findings suggest that, in general, circRNAs are expressed significantly lower in CRC cells compared to their normal cells counterparts (*P* = 8.7e-5; Figure S3). This observation is also in agreement with the findings of Zheng et al. (2016) and Bachmayr-Heyda et al. (2015) and suggest that this expression pattern indicates the role of circRNAs in CRC development. A positive expression relationship (although rare cases with negative relationship) was observed between circRNAs and their host genes based on this study (*P* < 2.2e-16). It is reasonable to suggest is that the lower expression of circRNAs in CRC cells may be responsive to lower expression of genes involving tumor depression.

Second, CRC circRNA sizes become smaller than that from their normal cell (Figure 2B). Similar trends were also observed in two prior studies on CRC circRNAs in which different CRC lines were used (Bachmayr-Heyda et al., 2015; Zheng et al., 2016). However, we also discovered the circularization score *H* of flanking introns of CRC circRNAs are significantly lower than that from their normal cell lines (Figure 2C), which was not reported previously. Therefore, we measured the circRNAs from both CRC and normal cells identified by Bachmayr-Heyda et al. (2015) and Zheng et al. (2016), respectively (Wilcoxon rank-sum test), and found same results in circRNA sizes (*P* < 2.2e-16 and *P* = 3.94e-7, respectively) but not in score *H* (Table S8). The lower score *H* may imply the main biosynthesis mechanisms of circRNAs in CRC cells are not based on reverse complementary sequences. And the changes of score *H* indicated that it is also a new aspect to study the mechanism of circRNA formation.

Particularly, in terms of sizes and score *H* of flanking introns of circRNAs, CRC metastasis circRNA sizes further become smaller than that from their primary tumor cells while score *H* of flanking introns of CRC metastasis circRNAs are also significantly lower than that from their primary tumor cells. This is the first observation for metastasis circRNAs and clearly additional studies in other CRC lines or other tumor tissues are merited to confirm the generality of this observation.

The characteristics of circRNAs in CRC cells and normal cells such as expression level, size and score *H* of flanking introns, indicates environmental inhibition of circRNAs biosynthesis in CRC cells in multiple perspectives.

### Development of circRNA biomarkers for CRC tissues and CRC metastasis

Previous studies have shown that circRNAs can be used as good biomarkers for tumor tissues (Wang X. et al., 2015; Ahmed et al., 2016; Kulcheski et al., 2016; Taborda et al., 2017; Zhang et al., 2017). In this study, we identified some circRNAs highly expressed only in CRC lines but not in normal lines or conversely. These circRNAs could serve as good measurable indicators for CRC development and metastasis. Based on expression patterns of circRNAs identified by this study, we can find some circRNAs highly expressed only in CRC SW480 and SW620 lines but not in normal NCM460 line (putative CRC biomarker) or only SW620 but not in SW480 and NCM460 (putative CRC metastasis biomarkers). By this way, top 12 circRNA biomarkers for CRC cells and top 15 biomarkers for CRC metastasis cells were selected and could be used for biomarker development in diagnosis of CRC in future (Table S9).

## Materials and methods

### Cell materials and cell culture

Normal human colon mucosal epithelium cells NCM460 (INCELL, San Antonio, Texas, USA), primary colorectal cancer cells SW480 and the lymph node metastatic variant cells SW620 were obtained from State Key Lab of Diagnostic and Treatment of Infectious Diseases, the First Affiliated Hospital, Zhejiang University School of Medicine. All media and supplements were purchased from Hyclone (Logan, UT, United States) and Sigma. Anti-CD63 (25682-1-AP) antisera was purchased from Proteintech. NCM460, SW480, and SW620 cells were cultured in DMEM medium supplemented with 10% FBS, 100 IU/mL penicillin-streptomycin at 37°C with 5% CO_2_.

### Metastatic ability *in vivo*

This study was carried out in accordance with the recommendations of Institutional Animal Care and Use Committee (IACUC) guidelines. The protocol was approved by the Laboratory Animal Welfare Ethics Committee, Zhejiang University (ZJU20170983). Six-week-old female Balb/C nu-nu mice were obtained from SHANGHAI SLAC LABORATORY ANIMAL CO. LTD (ShangHai, China), and maintained under specific pathogen-free conditions. Mice were injected as previously described (Hewitt et al., 2000). For intrasplenic injection (IS), 5 × 10^6^ cells were obtained and resuspended in 100 uL of PBS. Mice were anesthetized and a midline abdominal incision was made. The spleen was exteriorized via an abdominal midline incision and tumor cell suspension was injected slowly into the spleen. For assays of tumorigenesis and liver metastasis, mice were euthanized 4 weeks after injection and organs were weighed.

### RNA extraction and sequencing of three cell lines

Total RNAs from these three cell lines were extracted using RNeasy Mini Kit (QIAGEN). For preparation of RNA-Seq libraries enriched for circRNAs, total RNA was first treated with the Ribo-Zero rRNA Removal Kit (Epicenter) to remove rRNA according to the manufacturer's instructions, and then treated by RNase R (Epicenter) to deplete linear RNA. RNase R treatment was done by incubating 1 μg of rRNA-depleted total RNA with 5 U RNase R in 1 × RNase R buffer at 37°C for 30 min. RNA sequencing libraries (NCM460, SW480, and SW620) for Illumina Hiseq 3000 platform were constructed according to the manufacturer's instructions (Illumina). Three biological replicates were performed.

FastQC and fastx toolkit (Andrews, 2010; HannonLab, 2014) were used to check the quality of the sequencing reads and remove the adaptors and low-quality reads, respectively. RNA-seq and circRNA-seq data by this study have been deposited in GenBank under the accession number or Project PRJNA393626.

### Principle component analysis (PCA) of sequencing data

In order to evaluate the reproducibility of replicates, data of gene expression were used to do PCA. For each circRNA-seq and mRNA-seq sample, FASTQ reads were first mapped to human genome (GRCh38/hg38) obtained from Ensembl by STAR (Dobin et al., 2013). Then we used HTSeq-count (Anders et al., 2015) to count supporting reads of annotated genes and finally DESeq2 (Love et al., 2014) to draw PCA plots.

### Identification and annotation of circRNAs

For each circRNA-seq sample, FASTQ reads were first mapped to human genome (GRCh38/hg38) by BWA (Li and Durbin, 2009) and then CIRI2 (Gao et al., 2015) was used to identify circRNAs. The number of reads spanning back-spliced junctions was used as an absolute measure of circRNA abundance. While running CIRI2, default parameters were used for samples of cell lines.

According to the annotation of human genome (GRCh38/hg38) obtained from Ensembl (http://www.ensembl.org), all identified circRNAs were annotated. For each circRNA, we searched for transcript fragments that have overlap with the genomic position of circRNA and then defined the corresponding gene of this transcript fragment as the host gene of this circRNA.

### Calculation of circularization score *H* of circRNAs

In order to evaluate the characteristics of circRNAs from three cell lines (NCM460, SW480, and SW620). We used the algorithm presented by Ivanov et al. (2015) to calculate the score *H* of flanking introns of circRNAs. Only exonic circRNAs that come from middle exons of transcripts were used, based on the calculation principles of this algorithm. Therefore, in order to reduce the errors caused by total circRNA numbers, all score *H* have been weighted average adjusted by the total number of circRNAs identified in each cell lines.

### Expression quantification of circRNAs and protein-coding genes

For each circRNA-seq sample, DESeq2 (Love et al., 2014) was used to analyze the significant differences of gene expression among three cell lines (NCM460, SW480, and SW620). To estimate the relative expression of a circRNA, we normalized the number of reads spanning the back-spliced junction to the total number of mapped reads (units in billion) and read length. Therefore, the formula of a circRNA's relative expression is “number of circular reads/number of mapped reads (units in billion)/read length.” Here, we defined circRNAs with |log2 FC| > 1 and padj < 0.05 (*P*-value adjusted for multiple testing using Benjamini-Hochberg) as differential expressed circRNAs (DECs).

For each RNA-seq sample, FASTQ reads were first mapped to human genome (GRCh38/hg38) obtained from Ensembl by STAR (Dobin et al., 2013). Then we used HTSeq-count (Anders et al., 2015) to count supporting reads of genes and DESeq2 (Love et al., 2014) to analyze the significant differences of gene expression among three groups (NCM460, SW480, and SW620). We defined genes with |log2FC| > 1 and padj < 0.05 (*P*-value adjusted for multiple testing using Benjamini-Hochberg) as differential expressed genes (DEGs).

## Author contributions

WJ, LF, and HJ: Conceived and designed the experiments; XZ: Analyzed sequencing data; QC and LM: Analyzed the networks of circRNAs and genes; WJ, SL, LZ, XL, CL, and HJ: Performed biological experiments; WJ, XZ, and LF: Drafted and revised the manuscript; QC, CY, and MT: Revised the manuscript; All authors read and approved the manuscript.

### Conflict of interest statement

The authors declare that the research was conducted in the absence of any commercial or financial relationships that could be construed as a potential conflict of interest.
