# Supplementary material for: The Circular RNA Profiles of Colorectal Tumor Metastatic Cells
Source: Front Genet. 2018 Feb 9;9:34. doi: 10.3389/fgene.2018.00034 (PMC5811837; doi:10.3389/fgene.2018.00034)
Supplement: Supplementary file 10 [file Presentation1.pptx]

## Slide 1
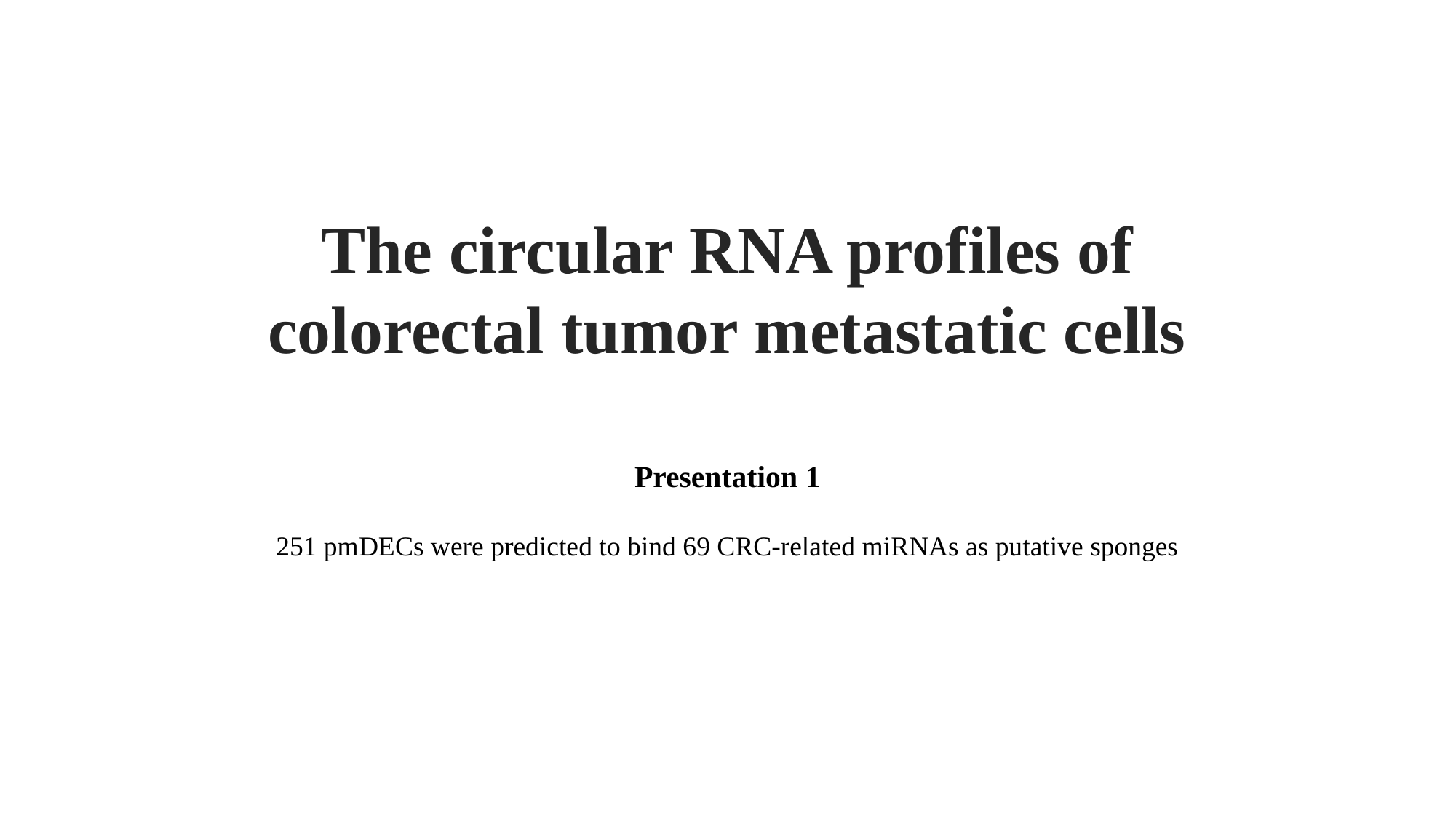

The circular RNA profiles of colorectal tumor metastatic cells
Presentation 1
251 pmDECs were predicted to bind 69 CRC-related miRNAs as putative sponges

## Slide 2
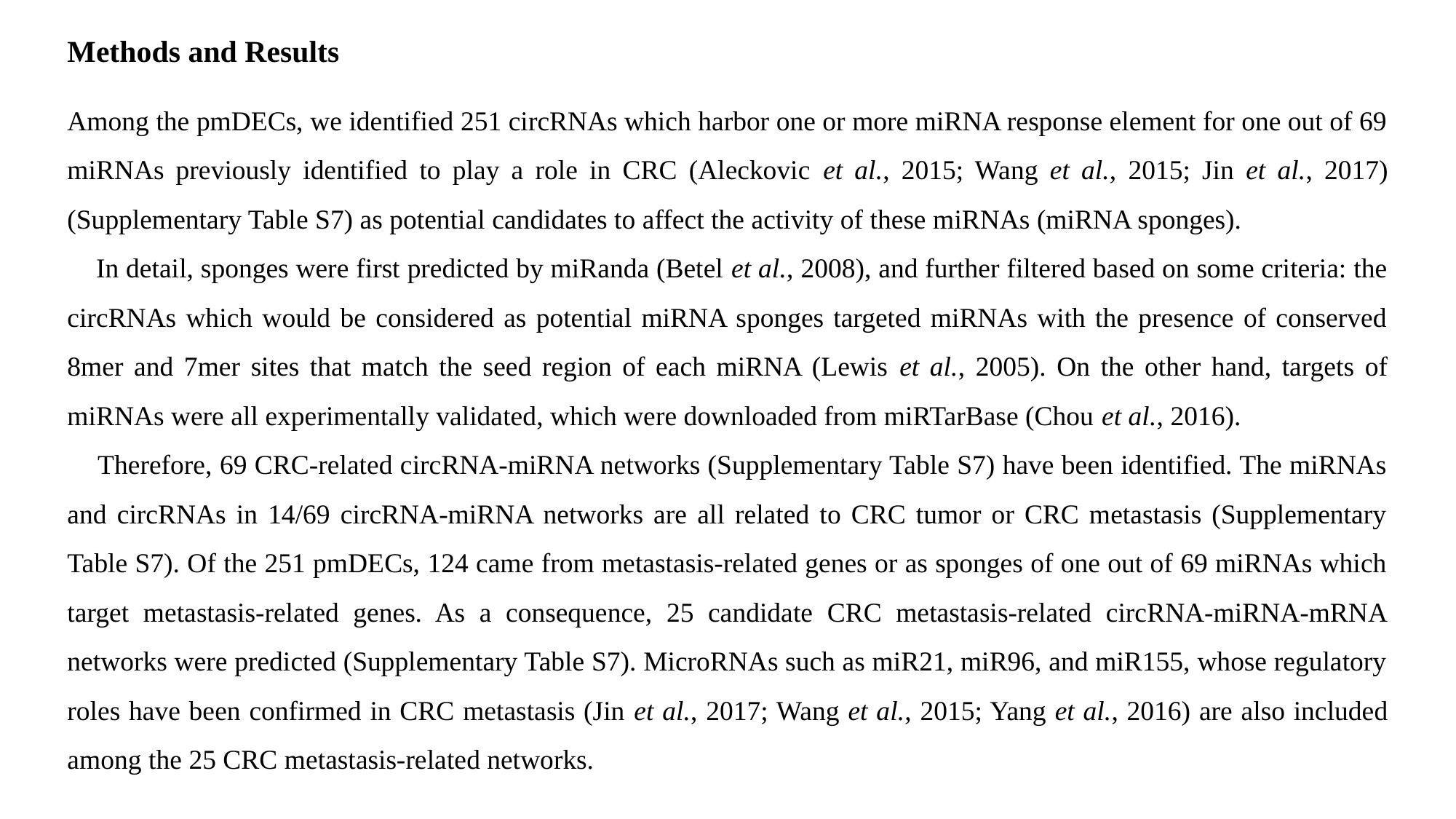

Methods and Results
Among the pmDECs, we identified 251 circRNAs which harbor one or more miRNA response element for one out of 69 miRNAs previously identified to play a role in CRC (Aleckovic et al., 2015; Wang et al., 2015; Jin et al., 2017) (Supplementary Table S7) as potential candidates to affect the activity of these miRNAs (miRNA sponges).
 In detail, sponges were first predicted by miRanda (Betel et al., 2008), and further filtered based on some criteria: the circRNAs which would be considered as potential miRNA sponges targeted miRNAs with the presence of conserved 8mer and 7mer sites that match the seed region of each miRNA (Lewis et al., 2005). On the other hand, targets of miRNAs were all experimentally validated, which were downloaded from miRTarBase (Chou et al., 2016).
 Therefore, 69 CRC-related circRNA-miRNA networks (Supplementary Table S7) have been identified. The miRNAs and circRNAs in 14/69 circRNA-miRNA networks are all related to CRC tumor or CRC metastasis (Supplementary Table S7). Of the 251 pmDECs, 124 came from metastasis-related genes or as sponges of one out of 69 miRNAs which target metastasis-related genes. As a consequence, 25 candidate CRC metastasis-related circRNA-miRNA-mRNA networks were predicted (Supplementary Table S7). MicroRNAs such as miR21, miR96, and miR155, whose regulatory roles have been confirmed in CRC metastasis (Jin et al., 2017; Wang et al., 2015; Yang et al., 2016) are also included among the 25 CRC metastasis-related networks.

## Slide 3
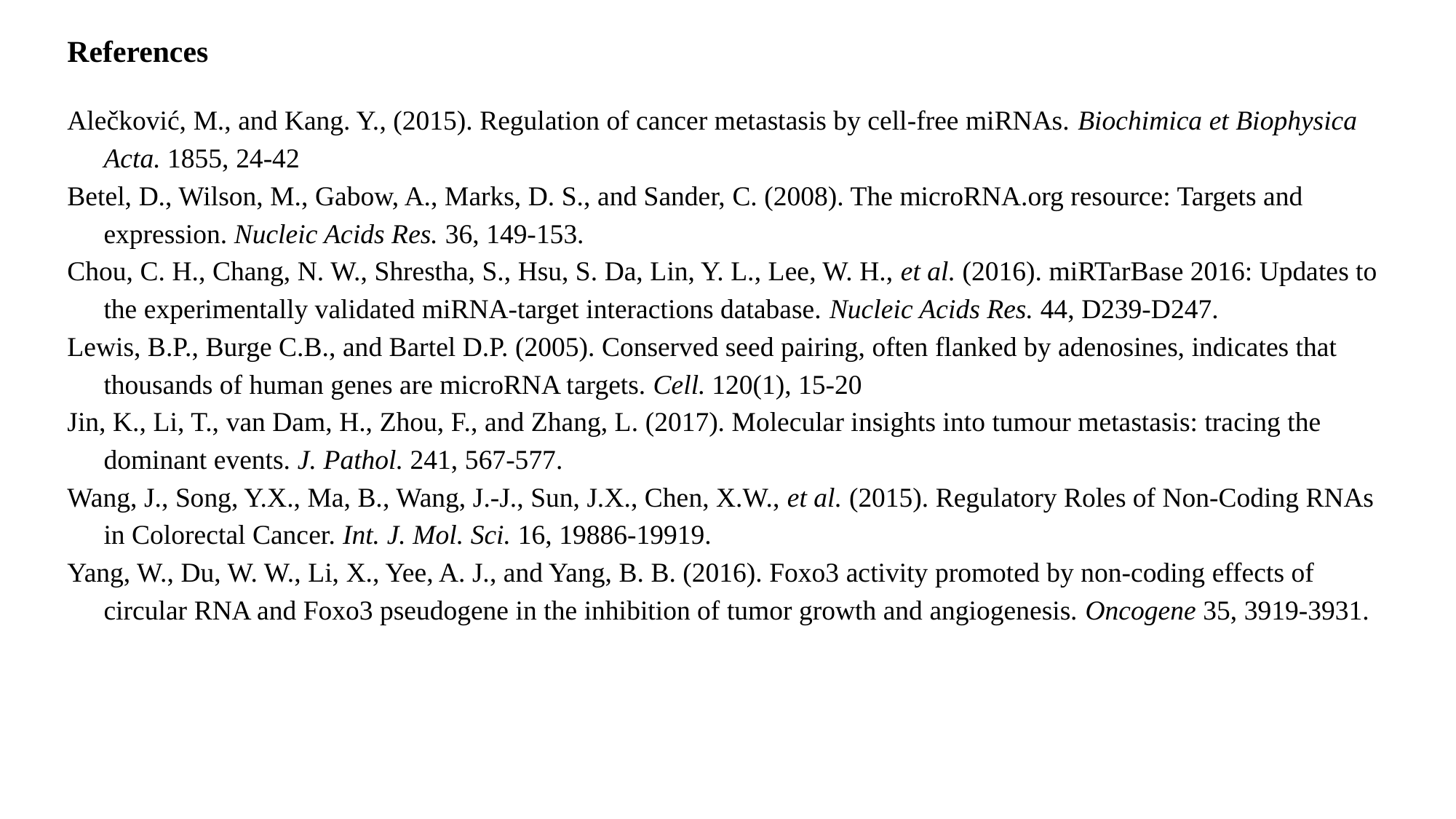

References
Alečković, M., and Kang. Y., (2015). Regulation of cancer metastasis by cell-free miRNAs. Biochimica et Biophysica Acta. 1855, 24-42
Betel, D., Wilson, M., Gabow, A., Marks, D. S., and Sander, C. (2008). The microRNA.org resource: Targets and expression. Nucleic Acids Res. 36, 149-153.
Chou, C. H., Chang, N. W., Shrestha, S., Hsu, S. Da, Lin, Y. L., Lee, W. H., et al. (2016). miRTarBase 2016: Updates to the experimentally validated miRNA-target interactions database. Nucleic Acids Res. 44, D239-D247.
Lewis, B.P., Burge C.B., and Bartel D.P. (2005). Conserved seed pairing, often flanked by adenosines, indicates that thousands of human genes are microRNA targets. Cell. 120(1), 15-20
Jin, K., Li, T., van Dam, H., Zhou, F., and Zhang, L. (2017). Molecular insights into tumour metastasis: tracing the dominant events. J. Pathol. 241, 567-577.
Wang, J., Song, Y.X., Ma, B., Wang, J.-J., Sun, J.X., Chen, X.W., et al. (2015). Regulatory Roles of Non-Coding RNAs in Colorectal Cancer. Int. J. Mol. Sci. 16, 19886-19919.
Yang, W., Du, W. W., Li, X., Yee, A. J., and Yang, B. B. (2016). Foxo3 activity promoted by non-coding effects of circular RNA and Foxo3 pseudogene in the inhibition of tumor growth and angiogenesis. Oncogene 35, 3919-3931.
