# Supplementary figures and images for: The Circular RNA Profiles of Colorectal Tumor Metastatic Cells
Source: Front Genet. 2018 Feb 9;9:34. doi: 10.3389/fgene.2018.00034 (PMC5811837; doi:10.3389/fgene.2018.00034)

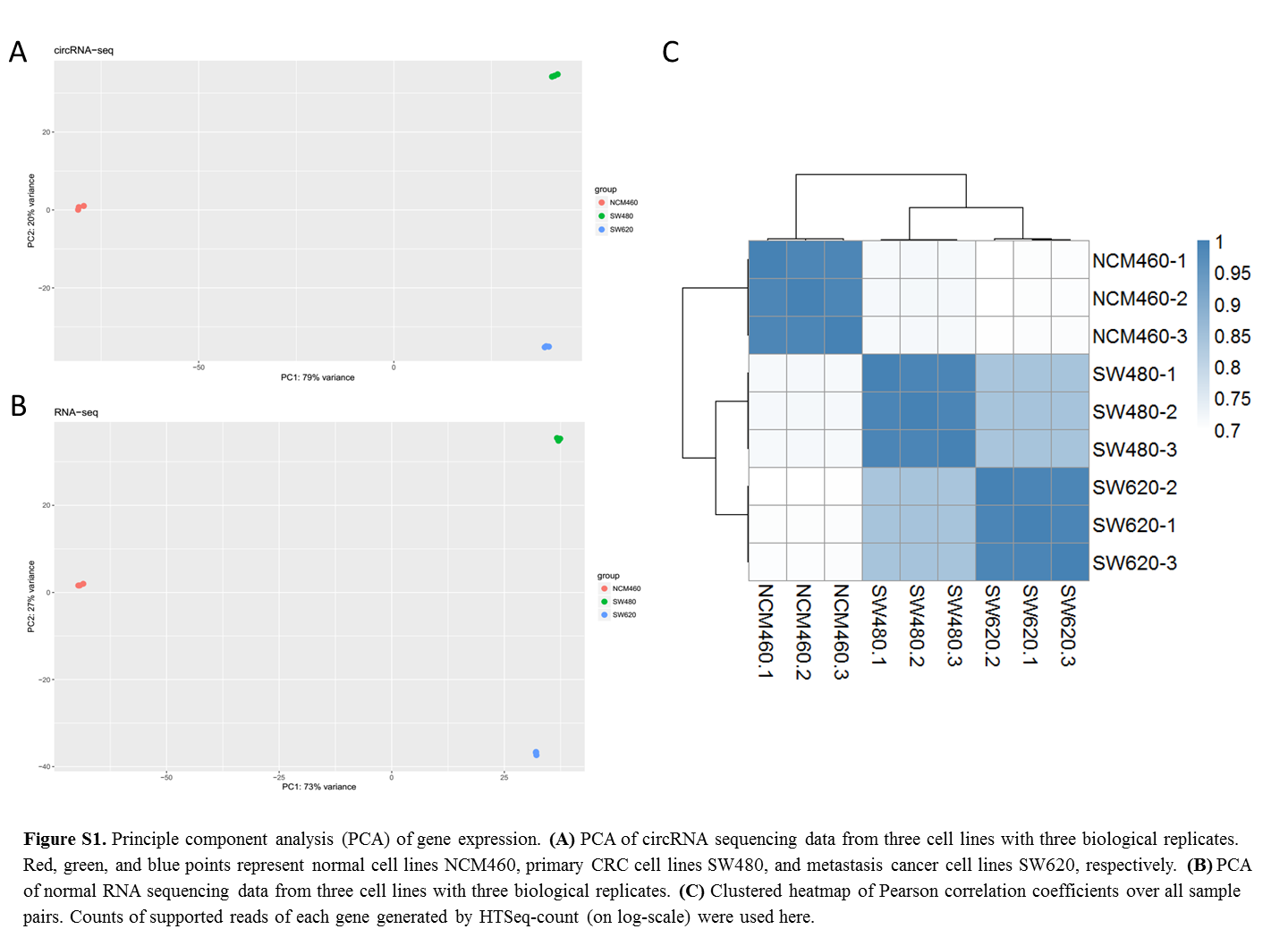

Supplement: Supplementary file 11 [file Image1.TIF]

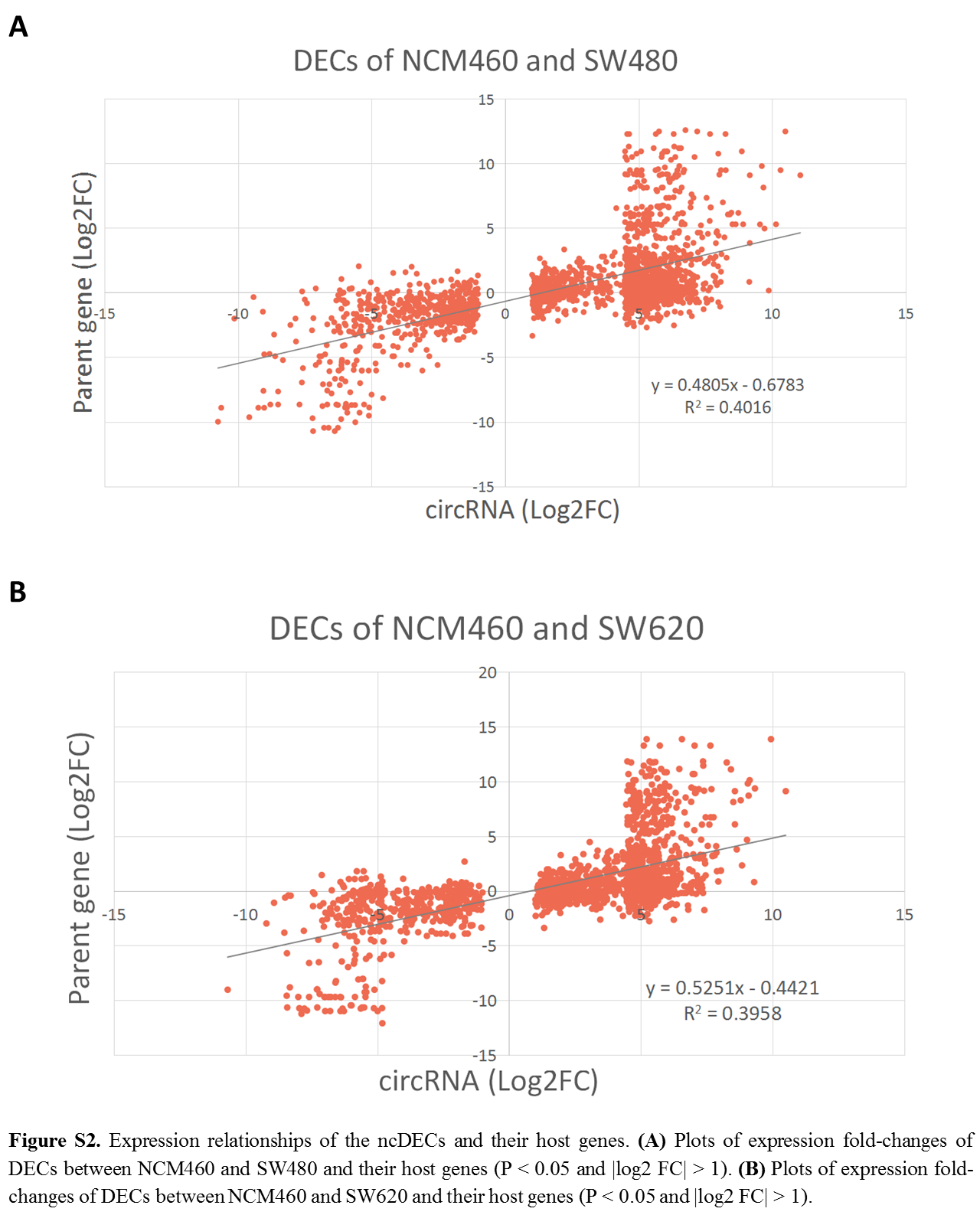

Supplement: Supplementary file 12 [file Image2.TIF]

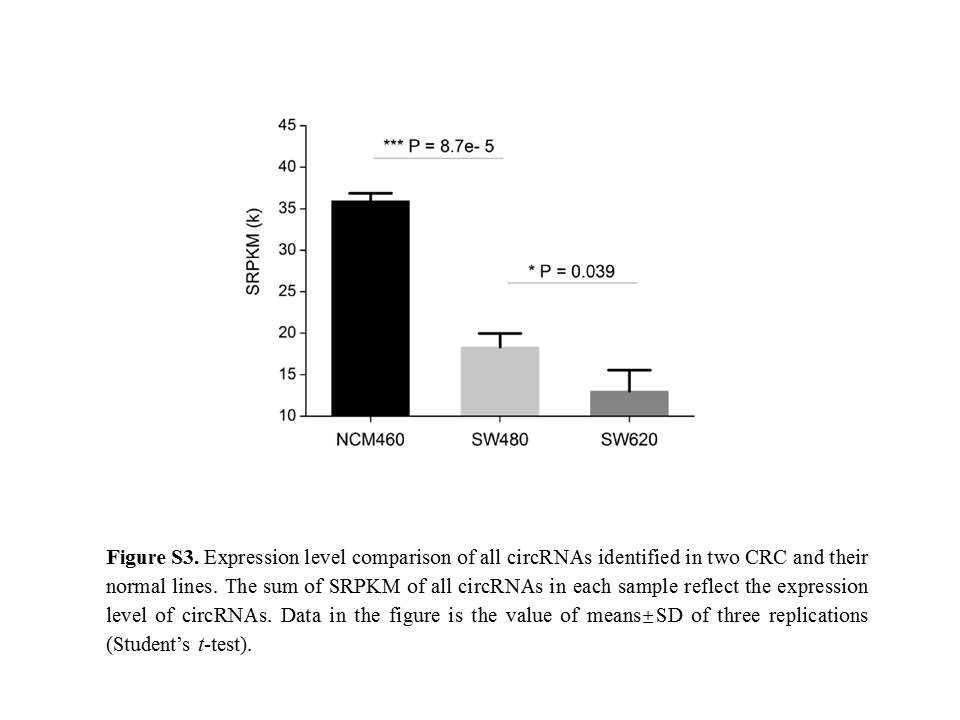

Supplement: Supplementary file 13 [file Image3.TIF]
